# Prevalence of Myopia in France: A Cross-Sectional Analysis

**DOI:** 10.1097/MD.0000000000001976

**Published:** 2015-11-13

**Authors:** Emilie Matamoros, Pierre Ingrand, François Pelen, Yacine Bentaleb, Michel Weber, Jean-François Korobelnik, Eric Souied, Nicolas Leveziel

**Affiliations:** From the Department of Ophthalmology, University Hospital of Poitiers (EM, NL); Epidemiology & Biostatistics, INSERM CIC 1402, University of Poitiers, Poitiers (PI); Ophtapointvision, Paris (FP, YB); Department of Ophthalmology, University Hospital of Nantes, Nantes (MW); Department of Ophthalmology, University Hospital of Bordeaux, Bordeaux (JFK); Department of Ophthalmology, Creteil Eye University (ES); and Inserm 1084, University of Poitiers, Paris, France (NL).

## Abstract

Refractive error (RE), particularly myopia, is the first cause of visual impairment throughout the world. This study aimed to depict the prevalence of myopia in a multicentric series of French individuals.

This cross-sectional analysis was carried out between January 2012 and November 2013 in eye clinics dedicated to REs. Data collection included age, gender, best-corrected visual acuity, RE, and any relevant medical history involving laser refractive surgery and cataract surgery. Exclusion criteria consisted of monophthalm patients or those with incomplete demographic data.

Prevalences in the overall population, by gender and by age groups were reported for mild myopia (−0.50 to −2.75 diopter [D]), moderate myopia (−3 to −5.75 D), high myopia (less than −6 D), and very high myopia (less than −10 D).

The analysis included 100,429 individuals, mean age 38.5 years (± 16.9). Overall prevalence of myopia was 39.1% (95% CI 38.8-39.4). Prevalences of mild, moderate, high and very high myopia were respectively 25.1% (95% CI 25.4-24.9), 10.6% (95% CI 10.4-10.8), 3.4% (95% CI 3.3-3.5) and 0.5% (95% CI 0.48-0.57).

Even if possible bias occurred in recruitment, our results are similar to RE data collected in nationally representative samples of Caucasians in other studies. This is to our knowledge, one of the largest European series of individuals dedicated to myopia prevalences in different age groups. These results confirm the importance of myopia as a major health issue in Western countries.

## INTRODUCTION

Myopia is an epidemic ocular disorder that has been increasing for the last 40 years.^1^ Environmental risk factors for myopia have been extensively investigated. A high level of education and near-work activities have been associated with higher prevalence, whereas outdoor activities could have a protective effect.^2^ Furthermore, different genetic factors with relatively small effect size have been reported in several genome-wide analyses, linkage analyses, and association studies in different populations, with more than 34 genes implicated in nonsyndromic myopia, most of them nonreplicated. These genes encode for various proteins located in extracellular matrix (collagen, fibromodulin, matrix metallopeptidase, catenin) or playing a role as growth factors (TGF-beta and hepatocyte growth factors) or as cholinergic and glutamate receptors.^3^ Eye growth and refractive development appear to be regulated by local signaling pathways within the eye from retina to sclera. Among different messenger molecules, experimental evidence supports the role of dopamine, which is released by dopaminergic amacrine cells, in the regulation of ocular axial growth and by consequence in the development of experimental myopia.^4,5^

Epidemiological studies on REs have shown differences between ethnic groups. Caucasians seem to present higher prevalence rates for myopia compared to African or Hispanic individuals and the highest rate has been reported among Asians.^6^

For example, the rate of myopia was reported to be 95% in student population among Chinese university students in Shanghai in 2009 and 82% in a Singaporean study of military conscripts in 2010.^7^

In the Caucasian population, the prevalence of myopia reported varies according to country, with a rate of 15% in Australia in 1994 versus 26% in the USA in 1990.^8,9^ The present study aimed at evaluating the prevalence of myopia in France in a large-scale clinical setting.

## PATIENTS AND METHODS

### Study Design

This study is a cross-sectional analysis of a large group of Caucasian individuals between January 2012 and November 2013, in 4 different eye treatment centers (2 in Paris, 1 in Lyon, and 1 in Bordeaux). These centers are especially dedicated to RE, and appointments are mainly given online.

The health interview and the visual examination were carried out by different physicians. They were started by orthoptists and pursued by ophthalmologists.

### Data Collection

The data collected included age, gender, any relevant medical history such as laser refractive surgery, cataract surgery, peripheral laser photocoagulation for peripheral retinal tears, and retinal detachment. All individuals underwent an ophthalmic examination applying the same procedure, involving noncycloplegic autorefraction on both eyes in adults (Tonoref, Nidek) and cycloplegic autorefraction with cyclopentolate for children. Best-corrected visual acuity (BCVA) was assessed on a Monoyer chart, a visual acuity decimal scale, and determined after objective autorefraction and subjective refinement by ophthalmologists. A slit lamp examination was performed to assess lens status (cataract or clear lens, pseudophakic, aphakic).

### Refractive Data

Myopia was defined as RE ≤−0.50 diopters (D), mild myopia as RE comprised between −0.50 and −2.75 D, moderate myopia as RE comprised between −3.00 and −5.75 D, high myopia as RE comprised between −6.00 and −9.75 D and very high myopia as RE of −10 D or less. Hyperopia was defined as RE ≥+0.50 D and emmetropia with RE comprised between −0.50 and +0.50 D. Refraction measurements were converted into spherical equivalents, calculated as the spherical value plus half of the astigmatic value. For homogenous comparisons of eye refraction data, analyzes were conducted on the right eye only and based on the subjective refraction as previously published.

Exclusion criteria included previous laser refractive surgery or cataract surgery. There was no particular systemic disorder, by either ethnicity or age, for exclusion criteria. Patients with incomplete data on right eye were automatically excluded from the analysis by the software.

The study adhered to the tenets of the Declaration of Helsinki. Data were declared to the “Commission Nationale d’Informatique et Liberté” (CNIL numbers 1695933, 1633782, 1705829, 1730110) and anonymized for study purpose.

### Statistical Analysis

Age and gender-specific prevalence of myopia, hyperopia, and emmetropia were assessed. Summary statistics are given as numbers (percentage). Statistical tests of homogeneity of prevalence across age groups and gender used the Chi-square test at the 5% significance level.

Because of the correlation of measures performed on both eyes and to ensure statistical independence between observations, only right eyes were analyzed. As a control, we checked the absence of significant difference between right and left eyes in patients when data were available for the 2 eyes.

## RESULTS

**FIGURE 1:**
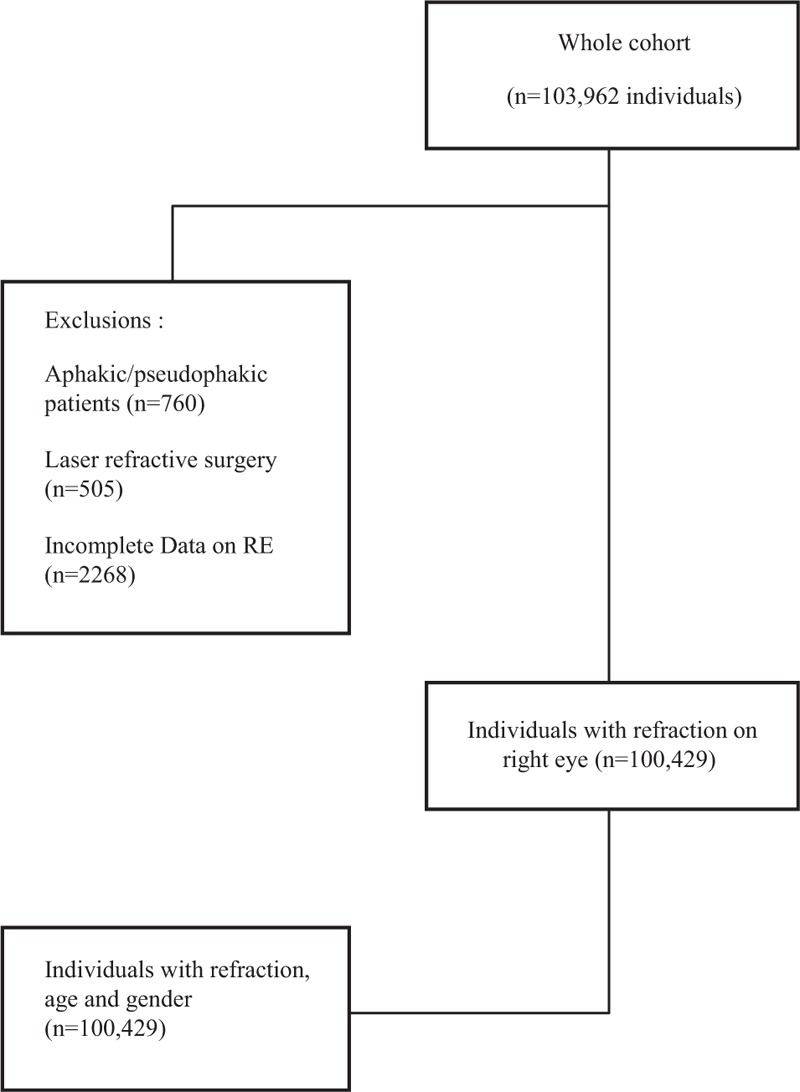
Flow chart of study patients.

A total of 100,429 individuals were initially included in the study. Out of this initial group, 102,705 patients were finally included in the analysis. The relevant data are presented in the flow chart (Fig. 1).

The mean age (SD) of individuals was 38.5 years (±16.9), with a sex ratio F/M of 1.39 (58,375 women /42,054 men).

**FIGURE 2:**
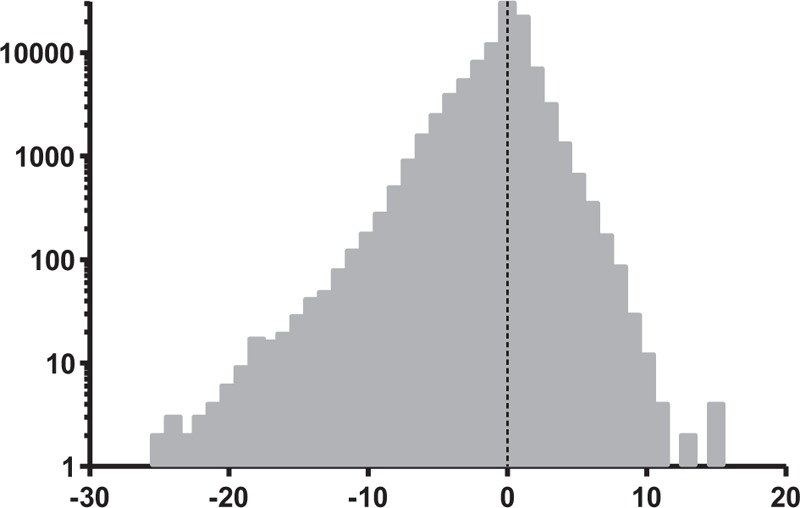
Distribution of refractive error on a semi-logarithmic scale in the whole series.

The RE range varied from −27.50 to +20.25 D, with a myopic shift more sizable than the hypermetropic shift. Figure 2 shows the distribution of RE on a semi-logarithmic scale in the whole series.

In this series, the overall prevalence of myopia was 39.1% (95% CI 38.8-39.4). The prevalences of mild, moderate, high and very high myopia were respectively 25.1% (95% CI 25.4-24.9), 10.6% (95% CI 10.4-10.8), 3.4% (95% CI 3.3-3.5) and 0.5% (95% CI 0.48-0.57).

### Myopia and Age

The age-specific prevalence of myopia and high myopia were higher in the 20- to 39-year olds with a rate of 52.4% (*P* <0.0001) and of 4.4 % (*P* < 0.0001) respectively. These data are more precisely indicated in Table 1.

**TABLE 1:**
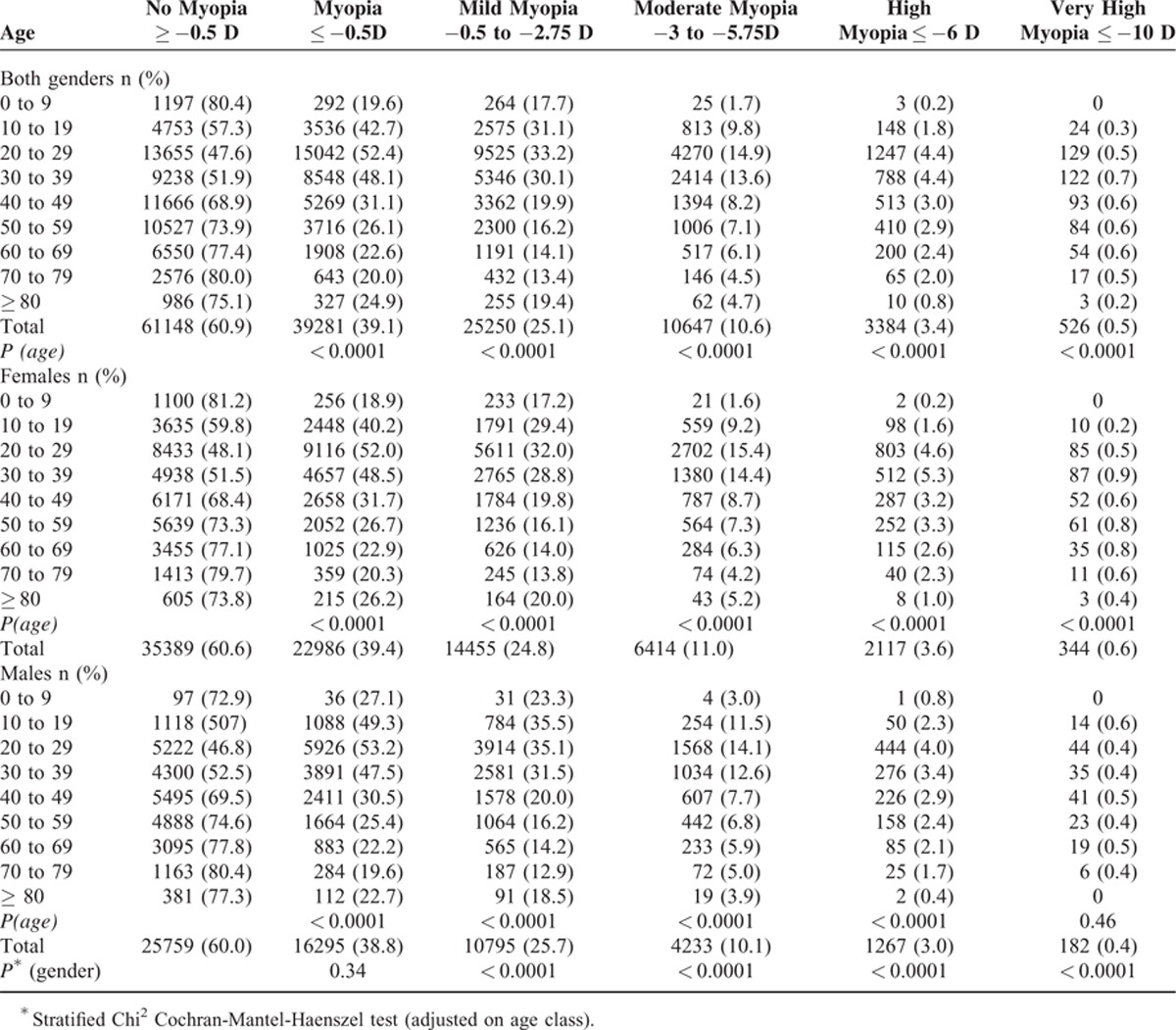
Prevalence of Myopia: Age by Gender-Specific Data

### Myopia and Gender

A total of 22,986 myopic women with a mean age of 33.5 (15.0) and of 16,295 myopic men with a mean age of 35.7 (14.4) were included. Prevalence of myopia was slightly higher in women than in men (39.4% vs.38.8%). Gender comparison for myopia showed that the prevalences of mild, moderate, high, and very high myopia were significantly higher among women than among men. These data are presented in Table 1.

## DISCUSSION

This study reported a prevalence of myopia of 39.1% in a large French group of individuals for whom refractive data were collected in eye clinics dedicated to REs. Some Caucasian and Asian studies have shown similar prevalences, as did the American NHANE Study with 42.6% of myopia^6^ and the Japanese Tajimi study with a rate of 41.8%.^10^ The prevalences of myopia in the main studies of the last 30 years are presented in Table 2.

**TABLE 2:**
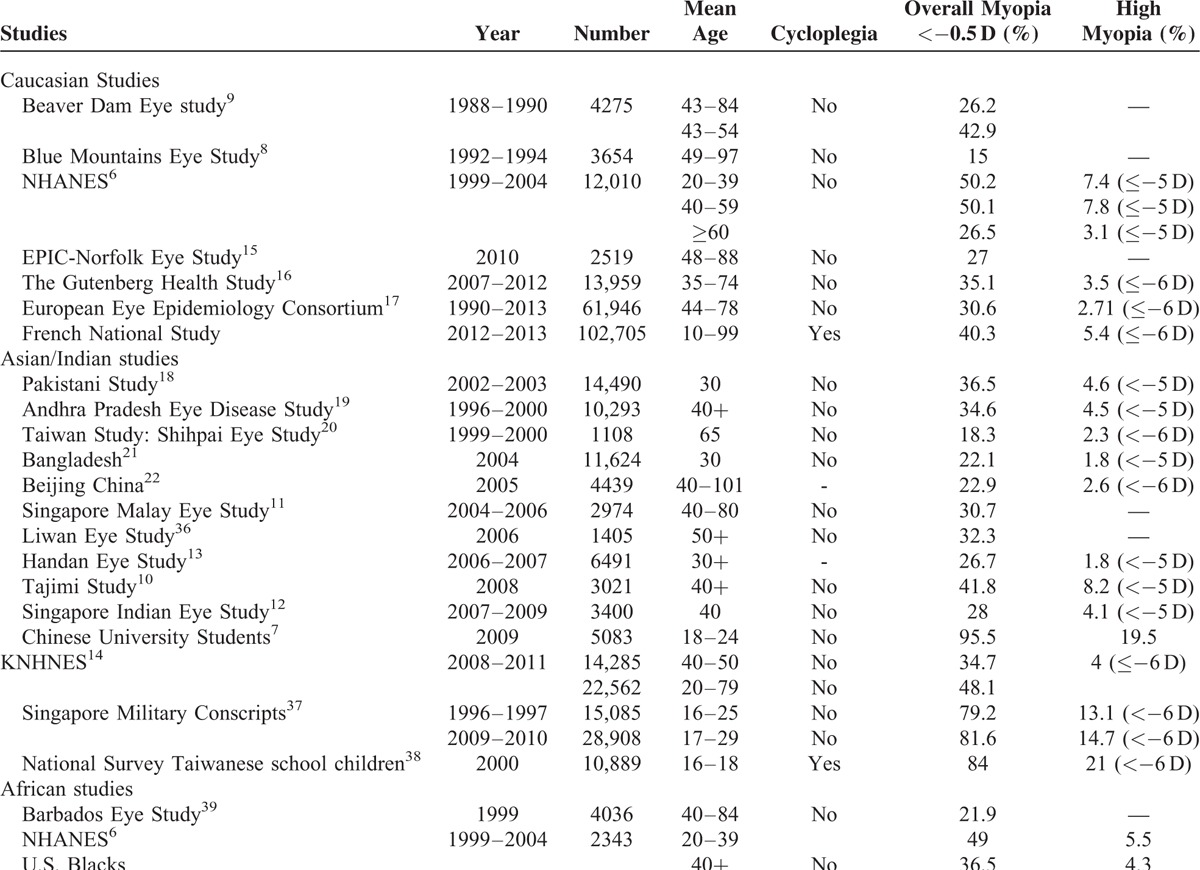
Prevalence of High Myopia in Different Ethnic Groups

### Myopia

In comparison with Asian studies, the prevalence of myopia in this French series was higher than the rates reported in the Handan Eye Study.^11–13^ However, the prevalence remained lower than in the Korean NHNE Study.^14^ Other studies based on a Caucasian population have also reported lower prevalences.^15,16^ A recent European meta-analysis likewise estimated a lower prevalence of myopia with a rate of 30.6%.^17^ These lower prevalences might be due to our definition of myopia, as most of the other studies defined this condition as RE <−0.5 D, while in our series myopia was defined as RE ≤−0.50 D. Furthermore, when comparing the prevalence of myopia in our study to this last study (39.1% vs. 30.6%) it is important to note that the mean age of participants in both studies (38.5 years vs. 62 years in the European Eye Epidemiology Consortium) is likely to explain this apparent discrepancy. Indeed, the peak of myopia usually observed in younger participants, reaching 47.2% in those aged 25 to 29 years in the European Eye Epidemiology Consortium and 52.4% in those aged 20 to 29 years in our cohort, finally showing similar results.

It is worthwhile to note that the recent prevalence of myopia has increased when compared to studies performed over recent decades in different ethnic groups in Australia (15%), in the USA with the BDE Study (26.2%), in Bangladesh (22.1%) and in China with the Beijing Study (22.9%) (Table 2).^8,9,21,22^

Even if discrete, the prevalence of myopia was higher among women than men in the present study (Table 1). Different studies have demonstrated that, even in animal models, myopia was more prevalent in the female gender.^23,24^

This difference could be due to hormonal factors as suggested in different studies.^25,26^

If the pathophysiology of myopia has not been clarified to date, we now know that it implies a disruption of the emmetropization process and a dysregulation of the axial length control. Thanks to animal models, the molecular mediators implicated in this process have been partially identified, including vasoactive intestinal peptide, dopamine, retinoic acid, glucagon, insulin, γ-aminobutyric acid, transforming growth factor, basic fibroblast growth factor, and insulin-like growth factor-1.^27–34^ As a complex disorder with environmental and genetic risk factors involving many inheritance modes, myopia has been associated with 261 genetic disorders.^35^

### High Myopia

The prevalence of high myopia in different ethnic groups is also reported in Table 2. In this French national series, the rate of high myopia was lower to that reported in the Japanese Tajimi Study (3.4% and 5.5%, respectively) and similar than in the GHS Study (3.3%) and in a Korean Study (34.7% and 4%, respectively).^9,14,16^ This discrepancy may be due to differences in terms of recruitment. Indeed, individuals recruited in the GHS Study were aged 35 to 74 years and individuals participating in the Korean Study were aged 40 to 50 years.

There is a lack of data on prevalence of myopia in the young population, mainly in Europe. Indeed, the most recent European studies reported values for older people aged 35 to 74 years or 44 to 78 years.^16,17^ In our study, when focusing on the prevalence of myopia in the 10- to 39-year range, we observed a rate of 49.5%. This prevalence was clearly inferior to the prevalence reported in the same population group in the Korean Study (75.1%).^14^

Most studies demonstrate an increase in the prevalence of myopia among younger urbanized individuals with generally higher myopia rates and longer axial length than in older individuals.^40^ This trend is likely to be explained by the environmental risk factor entailed by more near-work activities and fewer outdoor activities, both of which are frequently associated with a young urbanized and educated population.^41^ Conversely, the lower prevalence of myopia in rural population areas is likely to be due to reduced exposure to these factors.

## LIMITATIONS

The present study was not designed to assess the risk factors of myopia, because our main objective was to measure the prevalence of myopia in different population age groups, and not to focus on epidemiological risk factors that had previously been extensively studied.^14,15,41^ For this reason, we did not correlate prevalence of myopia to educational level or to other environmental factors.

We also acknowledge that the prevalence of myopia may have been underestimated in our study, by exclusion of pseudophakic individuals and refractive surgeries, but because the vast majority of our patients did not undergo refractive or cataract surgery, unshown data including these factors did not influence the overall prevalences of myopia and high myopia. On the other hand, because refractive data are not extracted from the general French population, our approach may overestimate the prevalence of myopia in individuals aged less than 50 to 55 years old, because people with no RE are less likely to make an appointment than people with RE. However, in groups aged 50 and more, our refractive data are likely to be comparable to those of the general French population because appointment for refraction is then required in case of presbyopia. Moreover, the prevalence of myopia observed in our study results is similar to a nationally representative sample of the a US population (NAHNES, n = 12,010 individuals) and to the European Eye Epidemiology (E(3)) Consortium (n = 61,946 individuals).^6,17^

Cycloplegia was not used systematically for adults, as it would have been considered unusual given the fact that in most studies on the same topic, it is not administered. Indeed, in a study including more than 2500 adult participants, the mean difference in spherical equivalent between measurements before and after cycloplegia was 0.29 D. The difference was greater among young persons with hyperopic REs.^42^ Considering children, the refractive data can be considered as reliable because cycloplegia with cyclopentolate was systematically used before refraction assessment.

This is to our knowledge one of the largest group of European individuals dedicated to REs. On this topic, international consortia involving a large number of individuals have focused on genetic factors of myopia, with the results of their studies published in a number of top-ranked journals.^43^ Given the complexity of myopic disease, in which genetic variants as well as environmental factors require careful consideration, there is a need for collaboration between different specialists in view of determining and depicting their respective causative effects.

Finally, regarding the high prevalence of myopia in our population, we cannot clearly confirm the hypothesis of a generally lower prevalence of myopia in European adults compared to Asian population.
